# Clinical Relevance of Soluble Forms of Immune Checkpoint Molecules sPD-1, sPD-L1, and sCTLA-4 in the Diagnosis and Prognosis of Ovarian Cancer

**DOI:** 10.3390/diagnostics12010189

**Published:** 2022-01-13

**Authors:** Janina Świderska, Mateusz Kozłowski, Katarzyna Nowak, Małgorzata Rychlicka, Dorota Branecka-Woźniak, Sebastian Kwiatkowski, Ewa Pius-Sadowska, Bogusław Machaliński, Aneta Cymbaluk-Płoska

**Affiliations:** 1 Department of Gynecological Surgery and Gynecological Oncology of Adults and Adolescents, Pomeranian Medical University in Szczecin, al. Powstańców Wielkopolskich 72, 70-111 Szczecin, Poland; jasia.swiderska@gmail.com (J.Ś.); kn13222@gmail.com (K.N.); aneta.cymbaluk@gmail.com (A.C.-P.); 2 Department of Nursing, Pomeranian Medical University, 71-210 Szczecin, Poland; mal_ryc71@wp.pl; 3 Department of Gynecology and Reproductive Health, Pomeranian Medical University, 71-210 Szczecin, Poland; dobrawo@gmail.com; 4 Department of Obstetrics and Gynecology, Pomeranian Medical University in Szczecin, al. Powstańców Wielkopolskich 72, 70-111 Szczecin, Poland; kwiatkowskiseba@gmail.com; 5 Department of General Pathology, Pomeranian Medical University in Szczecin, al. Powstańców Wielkopolskich 72, 70-111 Szczecin, Poland; ewapius@wp.pl (E.P.-S.); machalin@pum.edu.pl (B.M.)

**Keywords:** PD-1, PD-L1, CTLA-4, immune proteins, diagnostic biomarker, prognostic biomarker, ovarian cancer

## Abstract

It is crucial to find new diagnostic and prognostic biomarkers. A total of 80 patients were enrolled in the study. The study group consisted of 37 patients with epithelial ovarian cancer, and the control group consisted of 43 patients with benign ovarian cystic lesions. Three proteins involved in the immune response were studied: PD-1, PD-L1, and CTLA-4. The study material was serum and peritoneal fluid. The ROC curve was plotted, and the area under the curve was calculated to characterize the sensitivity and specificity of the studied parameters. Univariate and multivariate analyses were performed simultaneously using the Cox regression model. The cut-off level of CTLA-4 was 0.595 pg/mL, with the sensitivity and specificity of 70.3% and 90.7% (*p* = 0.000004). Unfavorable prognostic factors determined in serum were: PD-L1 (for PFS: HR 1.18, 95% CI 1.11–1.21, *p* = 0.016; for OS: HR 1.17, 95% CI 1.14–1.19, *p* = 0.048) and PD-1 (for PFS: HR 1.01, 95% CI 0.91–1.06, *p* = 0.035). Unfavorable prognostic factors determined in peritoneal fluid were: PD-L1 (for PFS: HR 1.08, 95% CI 1.01–1.11, *p* = 0.049; for OS: HR 1.14, 95% CI 1.10–1.17, *p* = 0.045) and PD-1 (for PFS: HR 1.21, 95% CI 1.19–1.26, *p* = 0.044). We conclude that CTLA-4 should be considered as a potential biomarker in the diagnosis of ovarian cancer. PD-L1 and PD-1 concentrations are unfavorable prognostic factors for ovarian cancer.

## 1. Introduction

Ovarian cancer is one of the most fatal malignancies in women. Epithelial ovarian cancer is a heterogeneous disease with histological subtypes that differ in cell and tissue origin, pathogenesis, gene expression, epigenetics, and prognosis. Due to the lack of screening tests for ovarian cancer and the presence of nonspecific symptoms or absence of symptoms in the early stages of ovarian cancer, this malignancy is detected in advanced clinical stages and continues to be a diagnostic challenge. Determination of blood markers such as CA125 (cancer antigen 125) and HE4 (human epididymis protein 4) is helpful in initial diagnostic management, although their levels may also increase in other pathologies. Transvaginal ultrasonography is also used in the primary diagnosis. There are also some algorithms, such as ROMA (Risk of Ovarian Malignancy Algorithm) or RMI (Risk of Malignancy Index), which are also used in the diagnosis of ovarian cancer [1,2]. Nevertheless, there is still a search for markers with sufficient sensitivity and specificity to be used as biomarkers in ovarian cancer diagnostics, especially to detect the cancer in its early clinical stage. Factors associated with ovarian cancer prognosis include performance status, the volume of residual disease after initial debulking surgery, and the FIGO stage [3]. The cytotoxic immune response, in which T-cells play a central role, is regulated by inhibitory and activating receptors. Immune checkpoints PD-1 and PD-L1 regulate the activation of cytotoxic T-cells [4]. Costimulatory molecules in immunoregulatory pathways take two forms of expression: the first is the membrane-bound form, while the second is the soluble form. The soluble form can be formed by two pathways. Thus, the soluble forms of different proteins are formed by two different pathways. The first pathway is the proteolytic cleavage of the membrane-bound form and the second pathway is the translation of alternatively spliced mRNA. Similarly, the presence of soluble forms of PD-1, PD-L1, and CTLA-4 was confirmed [5]. The role of the proteins studied is still unclear. Nevertheless, it was hypothesized that the binding of sPD-1 to mPD-L1/mPD-L2 can prevent mPD-1 from binding to PD-L1 and PD-L2, which counteracts the inhibitory effect of mPD-1 on T-cells [6]. In contrast, sPD-L1 was found to be a negative therapeutic and prognostic biomarker in several malignancies, such as renal cell carcinoma and multiple myeloma [5]. sCTLA-4 can inhibit the T-cell response. For this purpose, to prevent the binding of B7 costimulatory ligands to the CD28 costimulatory receptor in T-cells, they can bind to B7 on antigen-presenting cells [7]. To date, there are few studies on determining the utility of the soluble forms of the proteins we studied in ovarian cancer, so we emphasize all the more the relevance of our study. The treatment of ovarian cancer has not changed significantly for a number of years. Relatively recently, newer drugs have been added to treatment regimens with immune response molecules as their target points. Initially, they were used in successive lines of treatment. The first immunological drug used in ovarian cancer was bevacizumab (a recombinant humanized monoclonal antibody blocking angiogenesis by inhibiting VEGF-A (vascular endothelial growth factor A). Immune drugs with a different mechanism of action include immune checkpoint inhibitors [8]. Immune checkpoint inhibitors are not only used to treat ovarian cancer but also other malignancies, such as kidney and lung cancer [9]. After long-term clinical trials, attempts to introduce immunotherapy for the treatment of epithelial cancers focusing on the PD-1 (programmed death receptor 1)/PD-L1 (programmed death-ligand 1) pathway have begun to show increasingly promising results [10]. The EMA (European Medicines Agency) has approved PD-1 inhibitors (nivolumab and pembrolizumab) for the treatment of metastatic melanoma and other diseases such as non-small cell lung cancer and head and neck cancer [11,12]. Anti-PD-L1 agents, for example, durvalumab, avelumab, and atezolizumab, have also been approved by the U.S. Food and Drug Administration (FDA) for the treatment of recurrent or metastatic cancers of urothelial origin, Meckel cell carcinoma, non-small cell lung cancer, and squamous cell head and neck cancer [13]. Currently, the combination of anti-angiogenic agents with checkpoint inhibitors is expected to open a new era for better control of various epithelial cancers [14].

In this study, soluble PD-1, PD-L1, and CTLA-4 (cytotoxic T-cell antigen 4) are examined in serum and peritoneal fluid. The purpose of this study is to evaluate the clinical importance of the studied proteins as diagnostic and prognostic factors in ovarian cancer.

## 2. Materials and Methods

### 2.1. Participants

#### 2.1.1. Participation in the Study

Eighty female patients over the age of 18 years, hospitalized for ovarian cysts or tumors accompanied by peritoneal fluid between 2018 and 2020 in the Department of Gynecological Surgery and Gynecological Oncology of Adults and Adolescents, were enrolled in the study. Patients with a second primary malignancy, current acute inflammation, cirrhosis, renal failure, heart failure (NYHA > III), autoimmune thyroid disease, and collagenases have been excluded from the study. Each patient was thoroughly informed about the study. The women signed an informed patient consent to participate in the study. Depending on the ovarian pathology, the patients were divided into two groups. The study group consisted of 37 patients diagnosed with ovarian cancer (OC group). The control group consisted of 43 patients diagnosed with benign ovarian cystic lesions (BOC group). The presence of ovarian pathology was confirmed by imaging studies and histopathological examination.

#### 2.1.2. Characteristics of the Study Group

The mean age of patients was 58.5 years; among patients with ovarian cancer, it was 61.8 years, and among patients with benign ovarian cystic lesions, 55.6 years. Furthermore, patients with ovarian cancer were divided according to FIGO staging: 6 patients at stage I–II and 31 patients at stage III–IV. Regarding the histological malignancy of the cancer (grading), 9 patients had a low grade and 28 patients had a high grade. Patients with ovarian cancer were also divided based on the presence of ascites and primary therapeutic management. The detailed characteristics of the patients with epithelial ovarian cancer and benign ovarian lesions are shown in Table 1 and Table 2.

### 2.2. Instruments

#### 2.2.1. Pre-Laboratory Sample Preparation

During routine preoperative examinations, an additional 5 mL of whole blood was collected, which was immediately centrifuged and frozen to −80 °C. For primary surgery, blood was collected before surgery, whereas for neoadjuvant chemotherapy, blood was collected before the administration of chemotherapy. In addition, peritoneal fluid (approximately 6 mL) was collected during surgery and separated into two tubes. In patients who received neoadjuvant chemotherapy, peritoneal fluid was collected during the initial diagnostic laparoscopy. In the study groups, peritoneal fluid (in the form of ascites or fluid in the pouch of Douglas) was present in most patients. In patients who did not have peritoneal fluid, washing was performed and the fluid was collected to assess the presence of tumor cells and to determine the concentrations of the proteins we studied. This fluid was then processed by centrifugation at room temperature for 10 min (1000× *g* rotation) to remove impurities. The resulting material was frozen to −80 °C in two independent tubes. The material was then subjected to laboratory analyses.

#### 2.2.2. Laboratory Analysis—Multiplex Immunoassay

PD-1, PD-L1, and CTLA-4 concentrations were quantified in serum and peritoneal fluid in groups of patients with epithelial ovarian cancer and benign ovarian cystic lesion by multiplex fluorescent-bead-based immunoassays (Luminex Corporation, Austin, TX, USA) using commercial Human Immuno-Oncology Checkpoint Protein Magnetic Bead Panel 1 (Merck Millipore, Billerica, MA, USA). To the plate standard, control and samples (25 µL of each) were added together with multiplex antibody capture bead solution. Overnight, the plate was incubated on a shaker at 4 °C. Then, by using a hand-held magnet, each well was washed with 200 µL Wash Buffer 3 times. With a pipette, 25 µL of detection antibody cocktail was added to each well. Next, the plate was sealed and, for 1 h, incubated at room temperature with shaking. The next step was adding 25 µL streptavidin–phycoerythrin mixture to the plate and then subsequent incubation with agitation for half an hour in the dark. After performing the washing, the microspheres in each well were added to 150 µL Sheath Fluid and shaken at room temperature for 5 min. The plate was then read and, on the Luminex analyzer, analyzed, and analyte concentrations were determined from five different standard curves showing MFI (median fluorescence intensity) vs. protein concentration.

### 2.3. Statistical Analysis

Statistical analysis was performed using Statistica 10 PL software. To present a descriptive analysis characterizing particular groups and subgroups of patients, the following features were used: min, max, range, mean, and median. The distribution of patients was performed using available data and did not meet the criteria for parametric tests because of its heterogeneity. According to the above results, parametric tests could not be used, and all results were based on non-parametric significance tests. The Mann–Whitney U-test was used to compare two groups of female patients. Dunn’s post-hoc test was used to consider the relationship between the three groups. Due to the lack of normal distribution and heterogeneity of the group, Spearman’s rank correlation coefficient was used. An attempt was made to check whether the examined proteins meet the criteria of diagnostic markers. For this purpose, we used a ROC (receiver operating characteristic) curve and the calculation of the area under the curve to characterize the sensitivity and specificity of the studied parameters. Kaplan–Meier curves were used to illustrate the overall survival of patients. The log-rank test was used to assess the effect of the studied proteins on OS. Univariate and multivariate analysis using the Cox regression model was also performed simultaneously. The parameters of the multivariate Cox analysis included the age, FIGO staging, grading, and median concentrations of the study proteins. All analyses for study proteins were performed for both serum and peritoneal fluid concentrations. A value of *p* < 0.05 was considered an indicator of statistical significance.

## 3. Results

### 3.1. Serum and Peritoneal Effusion Fluid Concentration of Studied Parameters

The OC group had statistically significantly higher concentrations of all studied proteins compared to the BOC group, measured in both serum and peritoneal fluid (Table 3).

Comparison of the concentrations of the studied proteins was also performed in the OC group, taking into account staging. Serum PD-1 levels were found to be higher in patients with stage III–IV compared to stage I–II (*p* = 0.02717). Serum PD-L1 levels were also higher in patients with stage III–IV, but the statistical significance was borderline at *p* = 0.04992. Serum CTLA-4 levels, on the other hand, were higher in patients with stage I–II disease than in patients with stage III–IV, but the result was not statistically significant (*p* = 0.3329). CTLA-4 levels in peritoneal fluid were also higher in stage I–II patients compared to stage III–IV patients (*p* = 0.01259). PD-1 and PD-L1 concentrations in peritoneal fluid were slightly higher in stage I–II patients, but the results were not statistically significant (*p* = 0.88533, *p* = 0.74168, respectively) (Table 4).

The analysis of concentrations in the OC group was also performed, taking into account grading. However, there were no statistically significant differences in the concentrations of the studied proteins between the groups of patients with low-grade and high-grade ovarian cancer (Table 5).

### 3.2. Correlations between Studied Parameters

Spearman’s rank correlation was used to calculate correlations between concentrations of the proteins studied. We found six statistically significant correlations between the protein concentrations tested. We found a weak positive correlation between PD-1 (S) and CTLA-4 (S) (r = 0.352, *p* < 0.05), a weak negative correlation between PD-L1 (S) and CTLA-4 (S) (r = −0.352, *p* < 0.05), a moderate positive correlation between PD-1 (S) and PD-1 (PF) (r = 0.62, *p* < 0.05), moderate positive correlation between CTLA-4 (S) and PD-1 (PF) (r = 0.401, *p* < 0.05), moderate positive correlation between CTLA-4 (S) and CTLA-4 (PF) (r = 0.415, *p* < 0.05), and weak positive correlation between PD-1 (PF) and CTLA-4 (PF) (r = 0.334, *p* < 0.05). In addition, all other correlations between the studied proteins are shown in Table 6.

### 3.3. Receiver Operating Characteristic (ROC) Curve for Using PD-1, PD-L1, CTLA-4, CTLA-4/PD-L1, and PD-1/PD-L1 to Distinguish between Ovarian Cancer and Benign Ovarian Cystic Lesions

**Figure 1:**
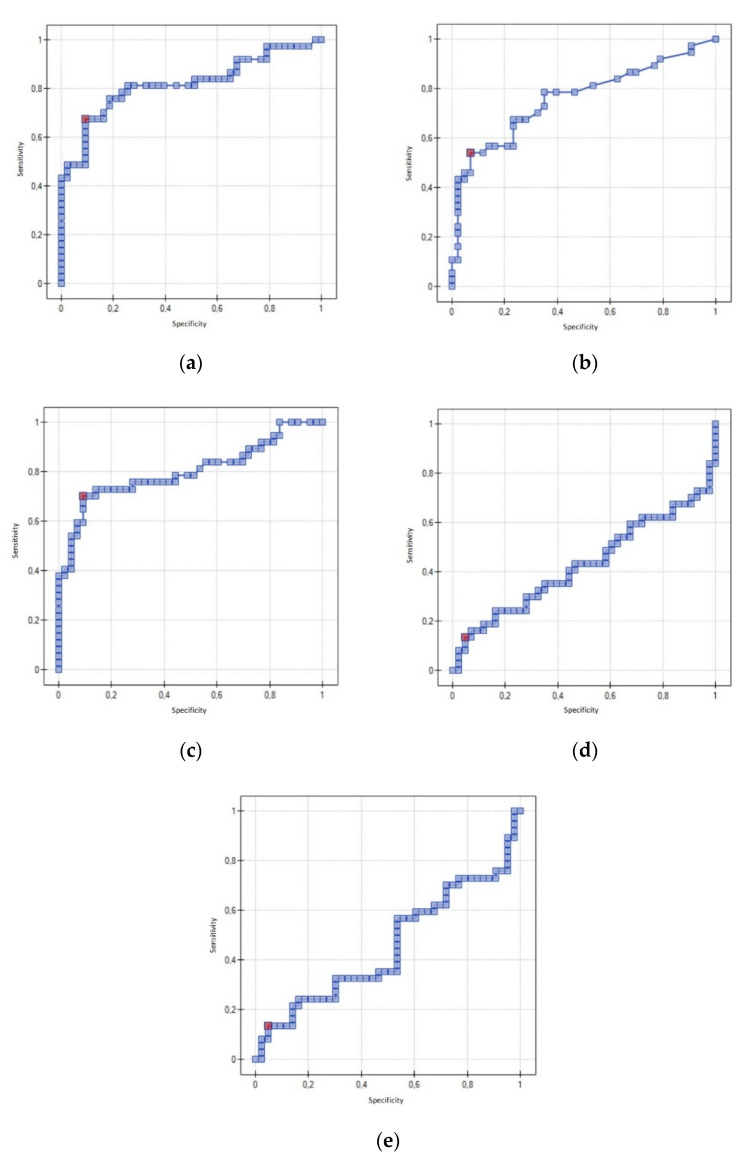
Receiver operating characteristic curve for using (**a**) PD-1, (**b**) PD-L1, (**c**) CTLA-4, (**d**) CTLA-4/PD-L1, and (**e**) PD-1/PD-L1 to distinguish between ovarian cancer and benign ovarian cystic lesions.

The cut-off values for PD-1, PD-L1, and CTLA-4 that were elevated in the serum of patients with ovarian cancer were calculated using ROC curve analysis. This analysis was performed to determine the utility of the proteins tested in the diagnosis of ovarian cancer. The analysis revealed that when the serum PD-1 concentration was 0.568 pg/mL or higher, the sensitivity and specificity were 67.6% and 90.7%, respectively (*p* = 0.000001). When the serum PD-L1 concentration was 0.459 pg/mL or higher, the sensitivity and specificity were 54.1% and 93%, respectively (*p* = 0.000066). When the serum CTLA-4 concentration was 0.595 pg/mL or higher, the sensitivity was 70.3% and the specificity was 90.7% (*p* = 0.000004). Using the data and subjecting them to statistical analysis, indices were created to account for the concentrations of two proteins. For CTLA-4/PD-L1, sensitivity was 13.5% and specificity was 95.3% (AUC 0.43, *p* = 0.273412). For PD-1/PD-L1, sensitivity was 13.5% and specificity was 95.3% (AUC 0.45, *p* = 0.484176) (Table 7). The ROC curves for PD-1, PD-L1, CTLA-4, CTLA-4/PD-L1, and PD-1/PD-L1 are shown in Figure 1.

### 3.4. Survival Analysis Using Kaplan–Meier Curve and COX Regression

**Figure 2:**
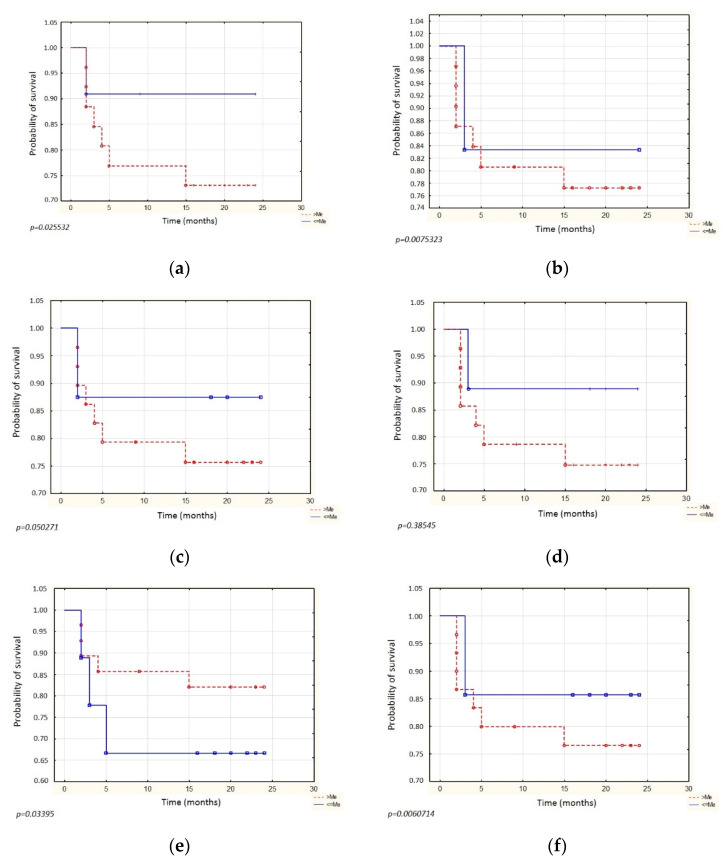
Kaplan–Meier curve representing overall survival for the following concentrations: (**a**) PD-L1 in serum, (**b**) PD-L1 in peritoneal fluid, (**c**) PD-1 in serum, (**d**) PD-1 in peritoneal fluid, (**e**) CTLA-4 in serum, (**f**) CTLA-4 in peritoneal fluid.

Survival analysis using the Kaplan–Meier curve and COX regression was performed to determine the utility of the proteins studied in the prognosis of ovarian cancer. Overall survival analysis using Kaplan–Meier curves was performed for median concentrations of study proteins in serum and peritoneal fluid. The follow-up time of the patients was 24 months. Around 91% probability of survival was observed in patients with serum PD-L1 levels below or equal to median compared to a survival probability of around 73% in patients with levels above the median (*p* = 0.025532). Similarly, there was a higher probability of survival for concentrations below or equal to the median of this protein in peritoneal fluid compared to concentrations above the median (approximately 83.5% vs. approximately 77.5%, *p* = 0.0075323). For serum PD-1 levels, the result was similar: the probability of OS for levels below or equal to median was approximately 87.5%, while above the median, it was approximately 75.5%, but there was borderline statistical significance (*p* = 0.050271). The result for the concentration of this protein in the peritoneal fluid was statistically insignificant (*p* = 0.38545). In contrast, survival for CTLA-4 concentrations was slightly different. Serum concentrations above the median had a higher probability of overall survival (approximately 82.5%) compared to concentrations below or equal to median (approximately 67%) (*p* = 0.033950). For CTLA-4 concentrations in peritoneal fluid, concentrations below or equal to median had a survival probability of approximately 85.5%, while concentrations above the median had a survival probability of 76.5% (*p* = 0.0060714) (Figure 2).

In univariant analysis, length of PFS (progression free survival) was affected by the staging, grading, and preoperative serum level of PD-L1 (*p* = 0.044, *p* = 0.031, *p* = 0.027, respectively). OS (overall survival) was affected by staging (*p* = 0.043), preoperative serum level of PD-1 (*p* = 0.039), and preoperative serum level of PD-L1 (*p* = 0.049). In multivariate analysis, independent risk factors affecting OS were age (*p* = 0.036), staging (*p* = 0.022), and PD-L1 level (*p* = 0.048). In contrast, independent factors affecting PFS were also staging (*p* = 0.048), PD-1 concentration (*p* = 0.035), and PD-L1 concentration (*p* = 0.016). A detailed presentation of the impact of the risk factors studied and the serum proteins studied is shown in Table 8.

Analyses were also performed for protein concentrations in peritoneal fluid. In univariant analysis, length of PFS was affected by staging (*p* = 0.047) and preoperative serum level of PD-L1 (*p* = 0.036). OS was affected by staging (*p* = 0.014), grading (*p* = 0.046), preoperative serum level of PD-1 (*p* = 0.033), and preoperative serum level of PD-L1 (*p* = 0.013). In multivariate analysis, the independent risk factors affecting OS were staging (*p* = 0.047) and PD-L1 level (*p* = 0.045). In contrast, the independent factors affecting PFS were age (*p* = 0.024), staging (*p* = 0.030), PD-1 concentration (*p* = 0.043), and PD-L1 concentration (*p* = 0.049). A detailed presentation of the effect of the risk factors studied and the proteins studied in the peritoneal fluid is shown in Table 9.

## 4. Discussion

Ovarian cancer is usually diagnosed at a very advanced stage [2]. Due to the uncharacteristic symptoms of ovarian cancer or their complete absence, it is important to search for new diagnostic methods that can be used to diagnose ovarian cancer at an earlier stage [15]. This study was designed to investigate whether PD-1, PD-L1, and CTLA-4 proteins measured in serum can be used to diagnose ovarian cancer. It was also investigated whether concentrations of these proteins might be risk factors for cancer development.

Firstly, the results showed that serum levels of all proteins in patients with ovarian cancer were statistically significantly higher than in patients with benign ovarian cystic lesions. Buderath et al. obtained similar results when it comes to sPD-L1, comparing 83 ovarian cancer patients with a group of 29 healthy women [16]. Another study published in 2017 had the same results for sPD-L1 levels, comparing cancer patients with healthy women or women with benign ovarian tumors. Interestingly, there was no significant difference in protein concentration between healthy women and those with benign ovarian tumors [17]. Changes in sPD-1 levels are observed not only in ovarian cancer but also in other malignancies. Goto et al., in a 2019 study, showed that sPD-1 level was found to be significantly higher in ovarian cancer patients and also non-small cell lung cancer, renal cell carcinoma, multiple myeloma, and acute myeloid leukemia patients compared to healthy individuals [18]. On the other hand, Gershtein et al. showed that sPD-L1 and sPD-1 levels did not differ significantly between the patients with ovarian cancer and the control group. In addition, they found that sPD-L1 levels in patients with benign tumors were significantly lower than in controls [19]. Furthermore, cut-off points were established for all proteins for which, at concentrations equal to or higher than the cut-off point, the specificity was high and, in all three cases, above 90% (PD1—90.7%, PD-L1—93%, CTLA-4—90.7%). The sensitivity of individual proteins was as follows: PD1—67.6%, PD-L1—54.1%, CTLA-4—70.3%. All the above results were statistically significant.

The researchers tried to create an index based on the concentrations of two proteins at the same time to check if combining two markers would give better results than using a single protein. This new marker could be used in clinical practice in the future as a modern auxiliary diagnostic tool in ovarian cancer. However, this did not produce the beneficial and expected results (for CTLA-4/PD-L1 AUC 0.43, *p* = 0.27; for PD-1/PD-L1 AUC 0.45, *p* = 0.48). Therefore, it appears that measuring individual proteins separately may have better clinical benefits than measuring the index that is based on the concentrations of two proteins.

On the basis of these results, it can be tentatively concluded that the determination of the concentrations of these proteins has a chance to be used in the diagnostics of ovarian cancer in the future. Due to the small size of the group tested in this study, we cannot conclusively determine whether the concentrations of these proteins should be used or not. A larger group of patients with ovarian cancer should therefore be investigated, and perhaps a new serum substance or substances could be identified for use in diagnostics.

Further results show that considering staging, serum levels of PD-1 and PD-L1 proteins are statistically significantly higher in ovarian cancer patients at stage III–IV than in patients with stage I–II. However, taking into account measurements of concentrations in peritoneal fluid, statistically significant results were obtained only for CTLA-4 concentration, which is higher in patients with stage I–II than in patients with stage III–IV. In the study conducted by Pawłowska et al., the plasma sPD-1 levels did not differ significantly between the different cancer stages, whereas with regard to sPD-1 concentration in peritoneal fluid, it was significantly higher in patients with stage III–IV cancer than in stage I–II patients [20]. In the described study, researchers tried to investigate whether there were differences in protein levels between the group of patients with low-grade ovarian cancer and high-grade ovarian cancer, but all the obtained results were statistically insignificant. In the 2020 study described above, considering grading, there were no significant differences in sPD-1 levels in either peritoneal fluid or plasma [20]. At this point, it is important to note the difference in group sizes in our study. At stage I–II, there were 6 patients; stage III–IV had 31 patients. In order to be able to draw concrete conclusions, it would be necessary to conduct a similar study in groups of similar size. However, these results may show a trend of increased levels of individual proteins in patients at different stages of disease.

Analyzing the Kaplan–Meier curves, it can be observed that for PD-L1 protein, if the result was less than or equal to the median, regardless of whether the measurement was performed in serum or peritoneal fluid, the probability of survival is higher than if the result was greater than the median (measurement in fluid: 2-year OS 91% versus 73%). Similar results were obtained in another study regarding ovarian cancer. It was shown there that both patients who were dead at the time of analysis and patients with disease progression had significantly higher sPD-L1 protein levels than patients who were alive or patients without disease progression [16]. In a French multicenter clinical trial on diffuse large B-cell lymphoma, researchers found that patients with high levels of PD-L1 protein have a poorer prognosis, with a 3-year OS of 76% versus 89% in healthy controls [21]. Regarding PD-1 protein, the results were similar; however, for the measurement in peritoneal fluid, the result was statistically insignificant (*p* = 0.39), and for the measurement in serum, the result was on the borderline of statistical significance (*p* = 0.05). Other researchers have shown that ovarian cancer patients with lower plasma levels of sPD-1 have longer 5-year survival than those with higher levels of the protein. In the same study, there was no statistically significant difference in OS of patients with different levels of sPD-1 in peritoneal fluid [20]. For CTLA-4 protein measured in peritoneal fluid, the relationship is similar to that for PD-L1. However, for CTLA-4 protein measured in serum, the relationship is reversed. If the score was above the median, then the probability of survival is higher. It is worth noting that the results obtained for PD-L1 proteins in serum and peritoneal fluid, PD-1 in serum, and CTLA-4 in peritoneal fluid are similar. Thus, for higher protein concentrations, the probability of survival is lower. An inverse relationship applies to CTLA-4 protein determined in serum. In this case, higher protein concentration is associated with better OS. This is a noteworthy relationship that would need to be explored in further future research. In addition, studies should be performed to investigate the reason for this different relationship for CTLA-4 protein measured in serum and peritoneal fluid.

The advantage of this study is the fact that the concentrations of the three proteins were also measured in the peritoneal fluid. This suggests that measurements of protein concentrations in both the peritoneal fluid and blood serum may be useful in identifying the group of patients with poorer prognoses, but further studies on the subject are needed.

In a study by Drakes et al., age was shown to be an independent prognostic factor in the survival of ovarian cancer. According to this study, patients over the age of 60 have a higher risk of death than patients diagnosed with cancer before the age of 60 [22]. In the same study, disease stage was shown to be another independent risk factor for ovarian cancer [22]. These findings are in agreement with our study. In addition to measuring the concentration of soluble proteins, their expression levels in tissues can also be studied. Such a study was conducted by Hamanishi et al., and it showed that PD-L1 expression in tumor tissue is an independent poor prognostic factor of ovarian cancer [23]. In our study, PD-L1 levels were found to be an independent risk factor for ovarian cancer, affecting both PFS and OS.

Studies on sPD-L1 have also been conducted in relation to other cancers than ovarian cancer, e.g., oral squamous cell carcinoma, nasal natural killer/T-cell lymphoma, and multiple myeloma [24,25,26]. Both OSCC and NNKTL patients had significantly elevated sPD-L1 levels [24,25]. MM patients with baseline lower levels of sPD-L1 had higher progression-free survival rates than patients with high levels of this protein [26]. Protein concentrations were also measured for colorectal cancer. The results showed that both elevated levels of PD-1 and CTLA-4 and co-elevated levels of these soluble proteins were correlated with poor overall survival and disease-free survival [27].

As mentioned above, researchers have also investigated the expression of proteins, not only their concentrations. Hamanishi et al. showed PD-L1 expression in the tumors of 88% of patients qualified for the study. In their study, it was shown that progression-free survival and overall survival of patients with high PD-L1 expression are significantly worse than those patients with a low expression of this protein. According to their study, patients without PD-L1 expression had a better prognosis [23]. Completely different results were obtained by Webb et al., who studied the expression of PD-L1 in high-grade serous ovarian cancer. In contrast to the results obtained by Hamanishi et al., they showed that PD-L1 is mainly expressed by tumor-associated CD68+ macrophages, not by tumor cells. PD-L1 expression was present in only 13.2% of HGSC tumors. They showed that the presence of PD-L1+ cells is prognostically favorable for patients with this type of cancer [28]. Another study showed that PD-L1 expression on tumor cells was associated with both better PFS and OS. Additionally, the presence of PD-1 on tumor cells was, according to this study, associated with better PFS. Moreover, a statistically significantly better prognosis was observed in high-density PD-1+ or PD-L1+ tumor-infiltrating lymphocytes (TILs), but only PD-1+ TILs appeared to have an independent prognostic impact on PFS and OS [29]. A similar study on breast cancer showed that the presence of PD-1+ TILs was associated with significantly worse OS and that the presence of such TILs was an independent negative prognostic factor of OS [30]. As for the results of CTLA-4 expression studies, they also vary widely between papers. Regarding ovarian cancer, CTLA-4 expression has a positive impact on OS and is associated with prolonged survival [31]. Yu et al. conducted a study on CTLA-4 expression in breast cancer. They showed that expression of CTLA-4 in lymphocytes improves prognosis while expression of this protein in tumor cells is associated with poor prognosis. According to their study, a higher density of CTLA-4+ interstitial lymphocytes is associated with better OS and DFS, whereas higher CTLA-4 expression on tumor cells is associated with shorter OS [32]. According to Paulsen et al., investigating CTLA-4 expression in non-small cell lung cancer, expression of this protein has a different prognostic significance for primary tumor versus lymph node metastasis of NSCLC. High CTLA-4 expression in stromal cells independently influenced significantly better disease-specific survival in the squamous cell carcinoma subgroup, while CTLA-4 expression in metastatic lymph nodes had an independent negative prognostic impact [33].

As can be seen, studies on PD-1, PD-L1, and CTLA-4 expression give completely different results. Therefore, the effect of the expression of these proteins on prognosis cannot be clearly stated, and further studies must be conducted.

In summary, as our study demonstrates, PD-1, PD-L1, and CTLA-4 can be used to help during the diagnostics of ovarian cancer. Based on AUC, sensitivity, and specificity, it seems that the best diagnostic marker that can be used for diagnostics of ovarian cancer among the proteins tested is sCTLA-4 measured in serum (sensitivity 70.3% and specificity 90.7%). Regarding prognosis, our study shows that for most of the studied proteins, the higher the protein concentrations, the lower the probability of survival. Only for CTLA-4 measured in serum, we observe an inverse relationship. Considering multivariate analysis, we show that PD-L1 levels in serum and peritoneal fluid are independent unfavorable prognostic factors affecting PFS and OS, and PD-1 levels measured in serum and peritoneal fluid are independent unfavorable prognostic factors affecting PFS. However, we believe that further research on the proteins mentioned above is needed to help conclusively determine whether any of them can be used in the diagnosis or prognosis of ovarian cancer.

## 5. Conclusions

CTLA-4 measured in serum should be considered as a potential biomarker in the diagnosis of ovarian cancer. Other tested proteins also have diagnostic potential, but further studies are needed. Considering Kaplan–Meier curves, favorable factors affecting OS are protein concentrations less than or equal to the median. Only for CTLA-4 protein measured in serum, a higher probability of survival occurs at concentrations greater than the median. Considering multivariate analysis, PD-L1 levels in serum and peritoneal fluid are independent unfavorable prognostic factors affecting PFS and OS. Furthermore, PD-1 levels measured in serum and peritoneal fluid are independent unfavorable prognostic factors affecting PFS.

## Figures and Tables

**Table 1:** Clinicopathological characteristics for the enrolled ovarian cancer patients.

Characteristic	Value	Number of Patients
FIGO staging	I–II	6
	III–IV	31
Grade	Low	9
	High	28
Ascites	Yes	29
	No	8
Primary therapeutic management	Surgery	26
	Neoadjuvant chemotherapy	11

**Table 2:** Mean age of the patients studied.

Characteristic	Age (Years)	Number of Patients
All patients	58.5 ± 12.5	80
Ovarian cancer	61.8 ± 12.4	37
Benign ovarian cystic lesion	55.6 ± 12.1	43

**Table 3:** Levels of serum and peritoneal fluid concentrations of the studied proteins in the OC group and the BOC group.

Characteristics		OC	BOC	*p*-Value
Serum level				
PD-1 [pg/mL]	Mean	8.42	1.98	0.000001
	Median	3.33	1.96	
	Range	0.73–50.08	0.71–5.13	
PD-L1 [pg/mL]	Mean	0.40	0.12	0.000067
	Median	0.30	0.07	
	Range	0.01–0.99	0.01–0.83	
CTLA-4 [pg/mL]	Mean	4.92	1.87	0.000004
	Median	4.00	1.84	
	Range	1.09–14.03	0.29–4.28	
Peritoneal fluid level				
PD-1 [pg/mL]	Mean	5.60	1.54	<0.000001
	Median	3.19	1.61	
	Range	1.02–27.10	0.26–3.13	
PD-L1 [pg/mL]	Mean	0.14	0.02	<0.000001
	Median	0.08	0.01	
	Range	0.01–0.85	0.00–0.13	
CTLA-4 [pg/mL]	Mean	5.58	2.14	<0.000001
	Median	4.23	1.98	
	Range	1.28–15.67	0.66–5.23	

**Table 4:** Levels of serum and peritoneal fluid concentrations of the studied proteins in the compared tumor FIGO stage groups.

Characteristics		Stage I–II	Stage III–IV	*p*-Value
Serum level				
PD-1 [pg/mL]	Mean	5.56	8.98	0.02717
	Median	2.52	4.64	
	Range	0.73–20.11	1.34–50.08	
PD-L1 [pg/mL]	Mean	0.21	0.44	0.04992
	Median	0.08	0.40	
	Range	0.01–0.87	0.01–0.99	
CTLA-4 [pg/mL]	Mean	6.46	4.62	0.3329
	Median	5.46	3.96	
	Range	1.21–13.45	1.09–14.03	
Peritoneal fluid level				
PD-1 [pg/mL]	Mean	5.93	5.54	0.88533
	Median	3.02	3.19	
	Range	1.83–19.97	1.02–27.10	
PD-L1 [pg/mL]	Mean	0.15	0.14	0.74168
	Median	0.08	0.08	
	Range	0.01–0.56	0.01–0.85	
CTLA-4 [pg/mL]	Mean	7.48	5.21	0.01259
	Median	6.82	4.2	
	Range	2.44–13.61	1.28–15.67	

**Table 5:** Levels of serum and peritoneal fluid concentrations of the studied proteins in the compared grading groups.

Characteristics		Low Grade	High Grade	*p*-Value
Serum level				
PD-1 [pg/mL]	Mean	5.79	8.89	0.43723
	Median	2.6	4.58	
	Range	1.74–20.11	0.73–50.08	
PD-L1 [pg/mL]	Mean	0.22	0.43	0.12421
	Median	0.08	0.4	
	Range	0.01–0.88	0.01–0.99	
CTLA-4 [pg/mL]	Mean	5.79	4.75	0.78453
	Median	3.72	4.10	
	Range	1.21–13.45	1.09–14.03	
Peritoneal fluid level				
PD-1 [pg/mL]	Mean	5.83	5.58	0.93472
	Median	3.02	3.19	
	Range	1.82–19.98	1.02–27.10	
PD-L1 [pg/mL]	Mean	0.14	0.14	0.62322
	Median	0.07	0.08	
	Range	0.01–0.57	0.01–0.85	
CTLA-4 [pg/mL]	Mean	7.22	5.25	0.2939
	Median	6.82	4.20	
	Range	2.44–13.62	1.28–15.67	

**Table 6:** The correlations between serum (S) and peritoneal fluid (PF) concentrations of PD-1, PD-L1, and CTLA-4 in OC patients, presented as the Spearman’s ranges‚ r correlation coefficient, *p* < 0.05 for underlined results.

Variable	PD-1 (S)	PD-L1 (S)	CTLA-4 (S)	PD-1 (PF)	PD-L1 (PF)	CTLA-4 (PF)
PD-1 (S)	1.000	0.173	0.352	0.62	0.02	0.152
PD-L1 (S)	0.173	1.000	−0.352	0.182	0.282	−0.243
CTLA-4 (S)	0.352	−0.352	1.000	0.401	−0.087	0.415
PD-1 (PF)	0.62	0.182	0.401	1.000	0.021	0.334
PD-L1 (PF)	0.02	0.282	−0.087	0.021	1.000	0.25
CTLA-4 (PF)	0.152	−0.243	0.415	0.334	0.25	1.000

**Table 7:** The diagnostic values of PD-1, PD-L1, CTLA-4, CTLA-4/PD-L1, and PD-1/PD-L1 for patients with ovarian cancer.

Marker	AUC (95% CI)	Sensitivity [%]	Specificity [%]	*p*-Value	Cut-Off Value [pg/mL]
PD-1	0.82 (0.72–0.92)	67.6	90.7	0.000001	0.568
PD-L1	0.76 (0.65–0.87)	54.1	93	0.000066	0.459
CTLA-4	0.8 (0.7–0.9)	70.3	90.7	0.000004	0.595
CTLA-4/PD-L1	0.43 (0.3–0.56)	13.5	95.3	0.273412	0.081
PD-1/PD-L1	0.45 (0.32–0.59)	13.5	95.3	0.484176	0.081

**Table 8:** Univariate and multivariate Cox regression model for serum concentrations of the proteins studied.

Univariate Analysis						
Variable	PFS			OS		
	HR	95% CI	*p*-Value	HR	95% CI	*p*-Value
Age (above vs. below median)	1.07	0.89–1.09	0.162	1.11	0.95–1.21	0.053
FIGO staging (III and IV vs. I and II)	1.16	1.06–1.21	0.044	1.09	0.97–1.15	0.043
Grade (high vs. low)	1.03	0.96–1.07	0.031	1.14	1.11–1.21	0.051
PD-1 level (above vs. below median)	1.13	1.06–1.20	0.056	1.12	1.08–1.18	0.039
PD-L1 level (above vs. below median)	1.26	1.24–1.29	0.027	1.13	1.09–1.15	0.049
CTLA-4 level (above vs. below median)	1.09	0.99–1.14	0.183	1.06	1.02–1.17	0.064
**Multivariate analysis**						
	**PFS**			**OS**		
	**HR**	**95% CI**	***p*** **-Value**	**HR**	**95% CI**	***p*** **-Value**
Age (above vs. below median)	1.10	1.07–1.12	0.052	1.14	1.08–1.15	0.036
Stage (III and IV vs. I and II)	1.19	1.14–1.22	0.048	1.16	1.12–1.18	0.022
Grade (high vs. low)	1.08	1.02–1.13	0.196	1.03	0.93–1.13	0.087
PD-1 level (above vs. below median)	1.01	0.91–1.06	0.035	1.08	0.98–1.10	0.061
PD-L1 level (above vs. below median)	1.18	1.11–1.21	0.016	1.17	1.14–1.19	0.048
CTLA-4 level (above vs. below median)	1.11	1.05–1.22	0.324	1.07	1.01–1.09	0.050

**Table 9:** Univariate and multivariate Cox regression model for concentrations of studied proteins in peritoneal fluid.

Univariate Analysis						
Variable	PFS			OS		
	HR	95% CI	*p*-Value	HR	95% CI	*p*-Value
Age (above vs. below median)	0.97	1.02–1.12	0.093	1.03	0.94–1.07	0.072
FIGO staging (III and IV vs. I and II)	1.06	1.01–1.12	0.047	1.07	1.02–1.08	0.014
Grade (high vs. low)	1.02	0.92–1.12	0.118	1.04	1.01–1.14	0.046
PD-1 level (above vs. below median)	1.04	0.99–1.08	0.201	1.12	1.08–1.16	0.033
PD-L1 level (above vs. below median)	1.06	0.99–1.11	0.036	1.05	1.00–1.07	0.013
CTLA-4 level (above vs. below median)	1.11	1.04–1.12	0.067	1.06	0.98–1.07	0.111
**Multivariate analysis**						
	**PFS**			**OS**		
	**HR**	**95% CI**	***p*** **-Value**	**HR**	**95% CI**	***p*** **-Value**
Age (above vs. below median)	1.09	1.06–1.14	0.024	1.12	1.06–1.15	0.144
Stage (III and IV vs. I and II)	1.23	1.19–1.26	0.030	1.16	1.15–1.18	0.047
Grade (high vs. low)	1.12	1.09–1.13	0.284	1.08	1.04–1.11	0.521
PD-1 level (above vs. below median)	1.21	1.19–1.26	0.043	1.16	1.12–1.18	0.110
PD-L1 level (above vs. below median)	1.08	1.01–1.11	0.049	1.14	1.10–1.17	0.045
CTLA-4 level (above vs. below median)	1.13	1.09–1.18	0.221	1.11	1.07–1.18	0.051

## Data Availability

The data presented in this study are available on request from the corresponding author, M.K., upon reasonable request.
